# Impact of COVID-19 on Older Adults: Changes in Health Access, Health, Socialization and Adaptive Coping Activities

**DOI:** 10.1093/geroni/igab046.2707

**Published:** 2021-12-17

**Authors:** Judith Scott, Stacy Yun, Sara Qualls

**Affiliations:** University of Colorado Colorado Springs, Colorado Springs, Colorado, United States

## Abstract

Indirect effects of stay-at-home guidelines may negatively affect mental health by reducing health self-care behaviors and engagement in social participation. This study reports on the impact of the COVID-19 pandemic on community-dwelling older adults’ perceived physical and mental health and everyday health behaviors. 126 older adults participated in a county-wide telephone survey during June-July of 2020, asking about changes in mental and physical health, and adaptations in health behaviors. We investigated the effects of three negative everyday health behavior changes during the pandemic (changes in health services access, perceived changes in health, and increased social isolation) as well as two positive everyday health behaviors (adherence to stay-at-home guidelines to reduce risk, and adaptive coping activities) on mental health and COVID-related distress. Examples of active coping strategies were stockpiling resources, spiritual practices, or outreach to others. Descriptive statistics, bivariate correlations, and multiple regressions characterized the impact of COVID-19 on perceived mental health. Descriptive data included changes in health service access, changes in mental and physical health, reduced social engagement, increased adherence to guidelines, and increased adaptive coping activities. Significant predictors of mental health impact of the pandemic were changes in health service access (β = .18, p < .05), health changes (β = .25, p < .01), and adaptive coping activities (β = .21, p < .05). Findings suggest COVID-19 distress may be alleviated with improved health care access and increased social contact. Mental health challenges may also benefit from increased engagement in adaptive coping activities.

